# Postoperative neuroleptic malignant syndrome-like symptoms improved with intravenous diazepam: a case report

**DOI:** 10.1007/s00540-013-1602-z

**Published:** 2013-04-04

**Authors:** Shun Kishimoto, Kumi Nakamura, Toshiyuki Arai, Ogino Yukimasa, Norihiko Fukami

**Affiliations:** Department of Anesthesia, Kyoto City Hospital, 1-2 Mibuhigashitakadacho, Nakagyo-Ku, Kyoto, 604-8845 Japan

**Keywords:** Neuroleptic malignant syndrome, Benzodiazepine withdrawal, Diazepam

## Abstract

A 75-year-old man who had undergone left upper lobectomy of the lung exhibited fever and insomnia on postoperative day (POD) 1 and muscle rigidity, autonomic instability, and somnolence on POD2 after epidural administration of droperidol and withdrawal of oral etizolam. He had not been known to have any neuromuscular diseases or psychiatric diseases, with the exception of anxiety disorder. Brain computed tomography did not show cerebrovascular disorders. Consultation with a neurologist led to a suspicion of neuroleptic malignant syndrome (NMS). Epidural droperidol was stopped and administration of dantrolene was initiated. These measures, in addition to supportive care, only partially ameliorated the symptoms of the patient, and consciousness disturbance developed; the patient finally became comatose on POD3. However, intravenous diazepam (10 mg) improved his symptoms abruptly. Subsequently, oral administration of lorazepam (1 mg/day) was started, and his symptoms disappeared within 2 days (POD5). Although NMS-like symptoms are rarely seen in clinical practice, some factors may induce it during the perioperative period, such as the administration of dopamine antagonists and the cessation of benzodiazepines. Intravenous diazepam is an effective treatment in cases with suspected gamma-aminobutyric acid (GABA) hypoactivity at the GABA_A_ receptor induced by the cessation of benzodiazepines.

## Introduction

Extrapyramidal symptoms caused by major tranquilizers is one of the common pathological states observed during the postoperative period. If it is observed with fever, mental state changes, and dysautonomia, the patient is suspected to have neuroleptic malignant syndrome (NMS). However, benzodiazepine withdrawal can also induce similar symptoms. Thus, when we seek for the pathogenesis of NMS-like symptoms of patients in the postoperative period, the drug history should be carefully taken into consideration because it might be the cause of the symptoms as well as anesthetics administered intraoperatively. In this report, we described a case of NMS-like symptoms that presented in postoperative period and improved with intravenous diazepam.

## Case report

A 75-year-old man (weight, 52 kg) diagnosed with primary lung cancer was scheduled for left upper lobectomy of the lung. He had undergone surgery for colon cancer under general anesthesia 22 years previously, without perioperative complications. He had not been known to have any neuromuscular diseases or psychiatric diseases, with the exception of anxiety disorder. He had been taking etizolam (1.5 mg/day) for the past 15 years, in addition to antihypertensive drugs. These drugs were discontinued on the day of operation.

In the operation room, after the placement of an epidural catheter, general anesthesia was induced using intravenous fentanyl (100 μg), thiamylal (250 mg), and rocuronium (50 mg), and was maintained using sevoflurane supplemented with fentanyl. The operation lasted 10 h and 28 min, which was unexpectedly long, because the tumor was highly adhesive to the surrounding tissue. Despite the long surgery, the quality of his emergence from general anesthesia was good; the patient responded to verbal commands and his trachea was extubated 15 min postoperatively. A continuous epidural infusion of fentanyl (16 μg/h), droperidol (0.1 mg/h), and ropivacaine (8 mg/h) was started, and the patient was transported to the intensive care unit (ICU) 30 min postoperatively.

On arrival at the ICU, the patient’s vital signs were normal and he responded to verbal commands, although he demonstrated hoarseness that was confirmed later to be caused by unilateral recurrent laryngeal nerve palsy, which was probably a result of the surgical procedures.

On POD1, he complained of insomnia and general fatigue. Because of the recurrent nerve palsy, his oral medications, including etizolam, were not given.

On POD2, he became somnolent and showed increased muscle tone. His skin surface was cold and diaphoretic. Respirations were almost normal, but he could not expectorate well. His blood pressure and pulse fluctuated from 139/82 to 197/116 mmHg and from 101 to 130 beats/min, respectively. His temperature rose to 37.6 °C, which responded to nonsteroidal antiinflammatory drugs. Serum creatine phosphokinase, AST/ALT, and lactate dehydrogenase levels rose from 85 U/l, 25/14 U/l, and 336 U/l to 1,757 U/l, 38/14 U/l, and 427 U/l, respectively. Myoglobinuria was not observed. Brain computed tomography did not show cerebrovascular disorders. A neurologist was consulted, and NMS was suspected as a result of droperidol administration. The epidural infusion of drugs, including droperidol, was ceased immediately and the administration of dantrolene (25 mg/day) was started via the nasogastric tube. Intravenous infusions of landiolol (5 mg/h), nicardipine (4 mg/h), and oxygen (via a face mask, 4 l/h) were started to keep his vital signs stable.

**Fig. 1:**
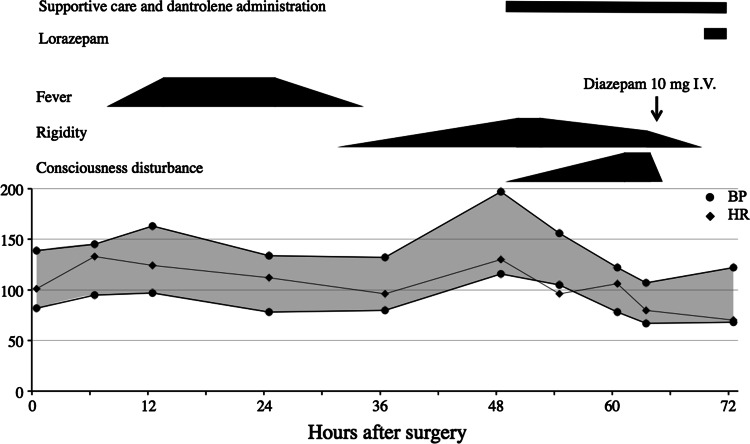
Clinical course of the patient: treatments, symptoms, and vital signs. *BP* blood pressure; *HR* heart rate. *Supportive care* includes intravenous infusions of landiolol (5 mg/h) and of nicardipine (1–4 mg/h) plus oxygen (via a face mask, 4 l/h)

On POD3, his muscle tone decreased slightly and the vital signs stabilized, but the patient was comatose and unresponsive to painful stimuli. Based on the suspicion of benzodiazepine withdrawal syndrome, a trial dose of a benzodiazepine was administered to the patient. The patient became responsive to verbal commands immediately after the injection of diazepam (10 mg). After 10 min, he could sustain a sitting position and told us about the hallucinations that he had had. Although he was still confused, excited, and had hallucinations, his mental status was dramatically improved (Fig. 1). Subsequently, oral administration of lorazepam (1 mg/day) was started and his symptoms disappeared within 2 days (POD5).

## Discussion

NMS is defined as a syndrome with hyperthermia, bradykinesia, rigidity, fluctuating consciousness, and autonomic dysfunction and is considered to be caused by dopamine hypoactivity. It can occur during the postoperative period as the result of the administration of major tranquilizers, including droperidol [1, 2]. This syndrome is lethal in 10 % of cases [3]; however, it is a self-limited disorder in most cases and frequently responds to conservative management after cessation of the drugs. Although hyperthermia is one of the major features of this syndrome, the maximum temperature can be less than 37.8 °C [4]. The treatment of NMS also includes the withdrawal of the suspected agent and the administration of dantrolene and bromocriptine.

Benzodiazepine withdrawal is known to induce various symptoms, including insomnia, anxiety, and convulsion [5, 6] and is usually treated with the administration of a benzodiazepine [7, 8]. Several cases have been reported in the literature in which NMS-like symptoms occurred during withdrawal of benzodiazepines [9, 10]. In this case, the patient had been medicated with etizolam, a thienodiazepine drug that is a benzodiazepine analogue, for 15 years until the day before surgery. Thus, benzodiazepine might be the main cause of these NMS-like symptoms.

The symptoms presented by the patient were compatible to NMS but not typical. If we pay attention to insomnia and general fatigue, which had been present before the NMS-like symptoms developed, we should consider that this clinical course might be caused not only by droperidol infusion but also by cessation of daily etizolam administration because these symptoms were more likely to be present in benzodiazepine withdrawal as “rebound insomnia” [11, 12].

In the current patient, although the treatments for NMS were effective for his muscle rigidity, autonomic dysfunction, and fever, they were not effective for his consciousness disturbance and bradykinesia. A trial dose of diazepam, however, dramatically improved the symptoms. Benzodiazepines are one of the options for the treatment for NMS [13], although it is not usually expected to have a great effect. Throughout the case, although the patient did not present the typical symptoms of NMS and benzodiazepine withdrawal, the great effect of diazepam implies that the pathogenesis of these symptoms were mainly caused by GABA_A_ receptor hypoactivity caused by the cessation of etizolam. Thus, we speculate that dopamine hypoactivity caused by droperidol induces NMS-like symptoms on the background of GABA_A_ receptor hypoactivity caused by etizolam cessation. If GABA_A_ receptor hypoactivity plays a major role in the development of these symptoms, benzodiazepine (as a GABA_A_ receptor agonist) should be the key treatment. In fact, there are case reports of NMS being responsive to diazepam [14, 15].

As the etiology of NMS is widely accepted as a syndrome caused by dopamine hypoactivity, it seems strange to administer a trial dose of benzodiazepine to a patient who presents NMS-like symptoms. However, in cases of perioperative benzodiazepine discontinuation, we can determine the contribution of GABA_A_ receptor hypoactivity to patients who exhibit NMS-like symptoms after general anesthesia via the administration of a benzodiazepine trial dose.
